# Maternal Exposure to Occupational Asthmagens During Pregnancy and Autism Spectrum Disorder in the Study to Explore Early Development

**DOI:** 10.1007/s10803-016-2882-6

**Published:** 2016-08-10

**Authors:** Alison B. Singer, Gayle C. Windham, Lisa A. Croen, Julie L. Daniels, Brian K. Lee, Yinge Qian, Diana E. Schendel, M. Daniele Fallin, Igor Burstyn

**Affiliations:** 1 Department of Epidemiology and Wendy Klag Center for Autism and Developmental Disabilities, Johns Hopkins University Bloomberg School of Public Health, 615 N. Wolfe St., Baltimore, MD 21205 USA; 2 Division of Environmental and Occupational Disease, California Department of Public Health, Richmond, CA USA; 3 Division of Research, Kaiser Permanente Northern California, Oakland, CA USA; 4 Department of Epidemiology, University of North Carolina Gillings School of Global Public Health, Chapel Hill, NC USA; 5 Department of Epidemiology and Biostatistics and the A.J. Drexel Autism Institute, Drexel University Dornsife School of Public Health, Philadelphia, PA USA; 6 Department of Public Health, Section for Epidemiology, Aarhus University, Aarhus, Denmark; 7 Department of Economics and Business, National Centre for Register-based Research, Aarhus University, Aarhus, Denmark; 8 Lundbeck Foundation Initiative for Integrative Psychiatric Research, iPSYCH, Aarhus, Denmark; 9 Department of Mental Health and the Wendy Klag Center for Autism and Developmental Disabilities, Johns Hopkins University Bloomberg School of Public Health, Baltimore, MD USA; 10 Department of Environmental and Occupational Health and the A.J. Drexel Autism Institute, Drexel University Dornsife School of Public Health, Philadelphia, PA USA

**Keywords:** Autism, Maternal occupation, Exposure, Maternal, Asthma, Allergy

## Abstract

Maternal immune activity has been linked to children with autism spectrum disorder (ASD). We examined maternal occupational exposure to asthma-causing agents during pregnancy in relation to ASD risk. Our sample included 463 ASD cases and 710 general population controls from the Study to Explore Early Development whose mothers reported at least one job during pregnancy. Asthmagen exposure was estimated from a published job-exposure matrix. The adjusted odds ratio for ASD comparing asthmagen-exposed to unexposed was 1.39 (95 % CI 0.96–2.02). Maternal workplace asthmagen exposure was not associated with ASD risk in this study, but this result does not exclude some involvement of maternal exposure to asthma-causing agents in ASD.

**Electronic supplementary material:**

The online version of this article (doi:10.1007/s10803-016-2882-6) contains supplementary material, which is available to authorized users.

## Introduction

Some evidence links increased risk of autism spectrum disorder (ASD) with maternal asthma and allergy around the time of pregnancy (Croen et al. 2005) and with maternal immune conditions in general (Comi et al. 1999; Keil et al. 2010; Sweeten et al. 2003). However, results across studies are inconsistent (Croen et al. 2005; Mouridsen et al. 2007; Micali et al. 2004; Lyall et al. 2014) and the mechanism by which maternal immune conditions are related to ASD is unclear. Epidemiologic studies also implicate some maternal environmental and occupational exposures capable of causing asthma and immune reactions, such as air pollutant exposures to acetaldehyde, formaldehyde, styrene, and metals (Leikauf 2002), in increasing risk of ASD, though associations have not been consistently reported across studies (Volk et al. 2011, 2013; Becerra et al. 2013; von Ehrenstein et al. 2014; Windham et al. 2006, 2013; Kalkbrenner et al. 2010; Roberts et al. 2007, 2013; McCanlies et al. 2012; Raz et al. 2015).

Asthmagens are agents known to trigger or exacerbate an asthmatic response. Examples include latex antigens, drug antigens, highly reactive chemicals (such as reactive dyes, amines, and biocides), and cleaning and disinfectant products. Exposure to asthmagens occur in a wide variety of occupations, including nurses, nursing aides, cleaners, carpenters, crop and animal producers, bakers, hairdressers, and beauticians (Kennedy et al. 2000). Some asthmagens cause asthma through immunologic pathways (e.g., latex antigens) whereas others act as irritants that result in airway damage (e.g., ammonia).

In studies assessing air pollutants in relation to ASD, exposure assessment is estimated based on census tract level modeled air pollutant concentrations (Windham et al. 2006; Kalkbrenner et al. 2010; Roberts et al. 2013) or measured pollutant exposures from air monitors closest to address at birth (von Ehrenstein et al. 2014). Occupational exposures are generally thought to be of greater intensity than general ambient environmental exposures and can be estimated on an individual level. McCanlies et al. (2012) linked ASD risk with parental report of occupational asphalt and solvent exposures and industrial hygienist’s assessment of occupational lacquer, varnish and xylene exposures. Based on occupational and industry information from birth certificates, a second study found higher odds of occupational exposures in general and specifically to exhaust and combustion products and disinfectants in mothers of children with ASD (Windham et al. 2013). Neither of these occupational studies focused specifically on ascertaining occupational exposures capable of triggering immune responses.

We sought to examine whether maternal exposure to occupational asthmagens during pregnancy influences risk of ASD in the offspring in a large multi-site case–control study, the Study to Explore Early Development (SEED), in order to contribute to a better understanding of modifiable risk factors for ASD. Since the effect of asthmagen exposure may differ depending on whether the mother has a history of asthma or allergy, we examined the association between occupational asthmagens and ASD among mothers with and without these conditions. Furthermore, because the prevalence of ASD is much higher in boys than girls (Fombonne 2003), we also examined child sex by occupational asthmagen exposure interactions.

## Methods

### Study Population

The Study to Explore Early Development is a multi-site case–control study (Schendel et al. 2012). The study was designed to examine phenotypes and co-morbidities related to ASD as well as genetic, lifestyle and environmental risk factors. The study consists of three groups of children between the ages of 30–68 months: children who meet the study criteria for ASD, children sampled from the general population (POP), and children with non-ASD developmental delays or disorders (DD). The SEED catchment area encompasses specific geographic locations in six different states: California, Colorado, Georgia, Maryland, North Carolina, and Pennsylvania. Eligible children were born in one of the catchment areas between September 1, 2003 and August 31, 2006, and residing in the same catchment area at the time of initial contact. Furthermore, eligible children were required to live with a knowledgeable caregiver who could communicate in English (or in English or Spanish in California or Colorado) (Schendel et al. 2012).

Children with possible ASD and DD were ascertained through multiple sources providing services for children with developmental disorders including hospitals, individual providers, clinics, and education and intervention programs. Parents with a child with an ASD or DD diagnosis could also contact the study directly to enroll. General population controls were ascertained through random sampling of vital records in the catchment areas (Schendel et al. 2012). Institutional review boards at each study site and at the Centers for Disease Control and Prevention (CDC) approved the SEED study. Informed consent was obtained from all enrolled participants.

### Outcome Assessment

Primary caregivers completed the Social Communications Questionnaire (SCQ) (Rutter et al. 2003a), a screener for autism spectrum disorder, during the study invitation phone call. Children with an SCQ score below 11 and without a previous ASD diagnosis were asked to participate in a general developmental evaluation in the clinic using the Mullen Scale of Early Learning (MSEL) (Mullen 1995). If the SCQ score was above 11, the child had previously received an ASD diagnosis, or a clinician suspected ASD during the clinic visit, the child additionally received a full ASD evaluation that included the Autism Diagnostic Observation Schedule (ADOS) (Lord et al. 1999, 2000; Gotham et al. 2007) and the Autism Diagnostic Interview Revised (ADI-R) (Lord et al. 1994; Rutter et al. 2003b). ASD was confirmed based on scores on the ADI-R and ADOS, as described in detail elsewhere (Wiggins et al. 2015).

We defined intellectual disability based on results from the MSEL, where a child was classified as having an intellectual disability if s/he had a Mullen Early Learning Composite Standard Score of less than 70.

### Occupational Exposure Assessment

Information on maternal occupational history was collected during a caregiver interview (CGI) administered as a computer assisted telephone interview shortly after enrollment in the study. Mothers were asked to report employment history corresponding to the period from 3 months before the start of the index pregnancy until breastfeeding ceased. If employed, mothers were asked to report each job held for 1 month or more in which they worked at least 10 hours per week. For each job meeting these criteria, mothers reported job title, employer, location, start and stop dates, hours worked per week, type of business, and main job duties.

All reported jobs were coded according to the International Labor Organization’s International Standard Classification of Occupations 1988 (ISCO-88) (International Labor Organization 1991) based on job title, task and industry information. Job coding was completed by the lead author (ABS). A second investigator (IB) reviewed ISCO codes and job texts for 370 of the 2708 jobs coded. Of these 370 jobs, 109 were selected for secondary review because the first coder had questions regarding the coding and the remaining 261 (10 % of the remaining jobs) were randomly selected for quality assurance. All discrepancies were resolved by reaching consensus and then applying any necessary changes to correct the rest of the coding. The agreement rate among the set examined for quality assurance was 92 % and we did not complete dual coding.

We used a previously developed asthma-specific job-exposure matrix (JEM) to estimate maternal occupational exposure to asthmagens during the pregnancy that was based on ISCO-88 job codes (Kennedy et al. 2000). This JEM is freely available (Kennedy and Le Moual 2011). Each job was classified “yes” or “no” as to whether or not the job typically has a high probability of exposure to an occupational asthmagen based on the standard categorization of the asthma JEM. The JEM was designed to favor specificity over sensitivity in exposure classification: when in doubt, a job was classified as unexposed. The exposure axis of the JEM consists of the following four subgroups of asthmagens with smaller classes of agents nested within: (1) high molecular weight agents (animals, fish, flour, plants, mites, enzymes, latex, bioaerosols, pharmaceuticals), (2) low molecular weight agents (highly reactive chemicals, isocyanates, cleaning/disinfecting products, wood dusts, and metals), (3) mixed environments (metal working fluids, textile, agricultural antigens), and (4) irritants. We completed two additional re-evaluation steps as recommended by the authors of the asthma JEM. These manual review steps are recommended because asthmagen exposures may differ among jobs with the same ISCO codes. The JEM authors included re-evaluation instructions in the JEM for specific ISCO job codes using information in the job descriptions. In the first step, the JEM authors specify reviewing selected ISCO job codes for possible job recoding. The step was generally designed either to help improve the precision of the job code or to give additional consideration to the industry information in cases where asthmagen exposure may differ based on industrial setting. In the second step, since asthmagen exposure may differ based on workplace tasks, selected ISCO codes are reviewed for possible asthmagen exposure group recoding following the instructions of the JEM authors. These steps were first completed by one investigator (ABS) and then reviewed by a second investigator (IB). All job coding and asthmagen exposure assessment was completed without knowledge of case or control status.

We restricted analyses to children whose mothers reported at least one job that overlapped with the pregnancy period. The approximate start date of the pregnancy was estimated by subtracting the gestational age of the child at delivery from the child’s date of birth. The gestational age was obtained from the birth certificate or imputed from medical record data if absent from the birth certificate. If the gestational age was missing from both the birth certificate and medical records, we estimated the approximate start date of the pregnancy by subtracting the mean gestational age for all children in SEED (265 days) from the child’s birthdate. We assumed that jobs started on the first day and ended on the last day of the job start and stop months, respectively. If we assessed that a mother was exposed at any pregnancy-period job then we counted the mother as exposed.

### Covariates

Information on potential confounders was obtained from the CGI and self-administered questionnaires which were completed by the mother/caregiver. Self-reported maternal race, maternal ethnicity (Hispanic or non-Hispanic), highest year of completed maternal education, total current household income, and parity were collected during the CGI. Maternal smoking (cigarettes) during the pregnancy was also ascertained from the CGI, including number of cigarettes smoked per day during each month of pregnancy. An active smoker was defined as a mother who either smoked one cigarette or more per day for at least 1 month of the pregnancy, or a mother who smoked less than one cigarette per day for at least 3 months of the pregnancy.

The maternal medical history questionnaire and the family autoimmune disease survey, two questionnaires mailed to study participants (Schendel et al. 2012), were used in concert with the CGI to create maternal medical condition variables. A mother was categorized as having allergy if, on the maternal medical history questionnaire, she reported having allergies diagnosed by a doctor with age of onset either before the birth of the child or unknown, or if during the CGI she reported a pregnancy-period medication for an allergic condition. A mother was categorized as having maternal asthma prior to delivery if a maternal asthma condition was reported on the autoimmune disease survey with an age of onset that was prior to the child’s delivery date or unknown, or if during the CGI she reported a pregnancy-period medication or treatment for asthma. Mothers were defined as having a history of psychiatric conditions based on reporting a history of attention deficit hyperactivity disorder, anxiety disorder, Asperger’s syndrome, autism, bipolar disorder, childhood disintegrative disorder, depression, obsessive compulsive disorder, personality disorder, pervasive developmental disorder, schizophrenia, self-injuring behavior, or suicide attempt in the maternal medical history form, or indicating a pregnancy-period treatment or medication for attention deficit hyperactivity disorder, anxiety disorder, bipolar disorder, depression, obsessive compulsive disorder, personality disorder, or schizophrenia in the CGI administration.

### Statistical Analysis

Only children whose mothers answered at least a portion of the occupational section of the CGI were eligible for this analysis (N = 2992 answered the occupational section of the CGI, including those who did not report a job during the pregnancy period). We excluded children with an incomplete case status classification (N = 247). Only one child per family was included in the analytic population (N = 42 excluded), leaving 2703 (685 ASD, 1054 DD, 964 POP). Children with an SCQ score below 11 and without a previous ASD diagnosis could be given a final classification of POP or DD based on ascertainment source, even if the child did not participate in the clinic developmental evaluation. Among the 2703 children, 78 DD and 87 POP children did not complete the clinic visit. Children of mothers who were unemployed during the pregnancy interval (N = 743) or where timing of occupation could not be determined (N = 38) were not considered in occupational exposure analyses.

Within each study group (ASD, DD, POP) we compared frequencies of demographic, socioeconomic, and maternal health characteristics in mothers considered exposed to mothers considered not exposed to occupational asthmagens during pregnancy. We used logistic regression to estimate the association between maternal occupational asthmagen exposure and ASD relative to the POP controls (and between DD and POP controls in separate supplemental analyses). Separate logistic regression models were fit for exposure categories in which more than 50 mothers cumulatively across the three study groups were exposed to: any asthmagen, any high molecular weight asthmagen, high molecular weight latex antigen, any low molecular weight asthmagen, low molecular weight highly reactive chemicals, and low molecular weight cleaning and disinfectant products. We also fit multiple logistic regression models to estimate these associations, adjusting for maternal race (white, black, Asian, Hispanic-race not specified, multi-racial/other), maternal education (less than high school, high school, some college/trade, bachelor’s degree, advanced degree), current household income at time of questionnaire (<$30,000, $30,000–70,000, $70,000–110,000, $110,000+), maternal age at birth (continuous), parity (1, 2, 3 or greater), active smoking during pregnancy (yes, no), maternal psychiatric condition history (yes, no), and child’s sex. These potential confounders were identified by consideration of factors likely associated with exposure and outcome that would not be potential mediators of an association between occupational asthmagen exposure and ASD. Analyses were restricted to families with complete covariate information. We completed additional sensitivity analyses where we classified mothers without pregnancy jobs as unexposed.

Since risk factors for ASD may differ by subtype of ASD, we also examined whether maternal occupational asthmagen exposure differed for ASD cases with and without intellectual disability compared to population controls. ASD children missing a Mullen Early Learning Composite score (n = 7) were excluded from this analysis.

We hypothesized that the association between occupational asthmagen exposure and ASD might differ depending on whether or not the mother had a history of asthma or allergy. As a result, among mothers with information on self-reported presence or absence of maternal asthma and allergy (441 ASD cases and 663 POP controls), we estimated two additional logistic regression models comparing ASD cases to POP controls, including interaction terms between any occupational asthmagen exposure and self-reported history of maternal asthma, or self-reported history of maternal allergy, prior to the birth of the index child. Since other studies suggest that the effect of environmental exposures on neurodevelopment may differ by the child’s sex, we also estimated a logistic regression model that included an interaction term between maternal occupational asthmagen exposure and child’s sex. We used criteria defined in Kaufman and MacLehose (2013) to evaluate evidence for heterogeneity of effects: evaluation of the interaction term.

## Results

Of the 2703 children enrolled in SEED from unique families with an ASD, DD, or POP classification and with some occupational interview data, 743 (27.5 %) mothers did not report working or having a job that overlapped with the pregnancy. For another 38 (1.4 %) mothers, we were unable to determine if there was a job that overlapped with the pregnancy (e.g. the start or end date of the job was missing, or the job start date was after the job end date). Mothers of POP controls were more likely to report having a job that overlapped with the pregnancy compared to mothers of children with ASD or DD (75.4 % of POP mothers vs. 69.2 % of ASD mothers and 68.4 % of DD mothers). Women with lower parity, higher education, higher income, older age at delivery, and of white, black, or multi-racial race or ethnicity were more likely to be employed during the pregnancy (data not shown). We restricted our analysis to the remaining 1922 (71.1 %) children whose mothers reported a job that we judged to overlap with the pregnancy. Among employed mothers, the average number of jobs per mother was 1.14, with 1681 (87.5 %) of these mothers reporting only one job during the pregnancy.

Among the 1866 children with employed mothers and complete covariate information (463 ASD cases, 710 POP controls, 693 DDs), we estimated that 260 (13.9 %) mothers had an occupational exposure to an asthmagen during the pregnancy. The most common asthmagen exposed jobs during pregnancy were nursing and midwifery professionals (27.2 % of asthmagen exposed jobs), institution-based personal care workers (11.0 %), hairdressers/beauticians (7.2 %), medical assistants (6.9 %), and veterinary assistants (4.8 %). Among POP control mothers, women with higher parity, lower levels of education, lower current household income and age 25–29 years old at the time of the index child’s birth were more likely to work in a job with exposure to at least one occupational asthmagen (Table 1). Among POP controls, the likelihood of exposure was higher among Black, Hispanic, or multiracial/other mothers (14.3, 18.8, and 21.6 % exposed, respectively) than among mothers who classified themselves as White or Asian (12.9 and 4.3 % exposed, respectively). The prevalence of occupational asthmagen exposure was 10.6 % in mothers with a self-reported history of allergy compared to 15.9 % in those without a self-reported history of allergy among controls (Table 1).

**Table 1:** Maternal occupational asthmagen exposure by characteristics among population controls (POP) and ASD cases (ASD)

Characteristics	POP, N (% exposed)		ASD, N (% exposed)	
	Unexposed (N = 615)	Exposed (N = 95)	Unexposed (N = 384)	Exposed (N = 79)
Child’s sex				
Female	287	48 (14.3)^a^	47	17 (26.6)^b^
Male	328	47 (12.5)	337	62 (15.5)
Parity				
1	330	42 (11.3)	218	37 (14.5)
2	206	35 (14.5)	113	26 (18.7)
3 or greater	79	18 (18.6)	53	16 (23.2)
Maternal race				
White	467	69 (12.9)	244	44 (15.3)
Black	84	14 (14.3)	83	20 (19.4)
Other^c^	64	12 (15.8)	57	15 (20.8)
Maternal education				
High school or less	43	11 (20.4)	37	18 (32.7)
Some college/trade	134	27 (16.8)	120	31 (20.5)
Bachelor’s degree	227	32 (12.4)	124	19 (13.3)
Advanced degree	211	25 (10.6)	103	11 (9.6)
Current household income				
<$30,000	68	17 (20.0)	79	25 (24.0)
$30,000–70,000	149	19 (11.3)	93	26 (21.8)
$70,000–110,000	170	26 (13.3)	112	15 (11.8)
≥$110,000	228	33 (12.6)	100	13 (11.5)
Maternal age at birth				
<25 years old	59	8 (11.9)	45	11 (19.6)
25–29 years old	137	32 (18.9)	87	20 (18.7)
30–34 years old	239	32 (11.8)	138	25 (15.3)
≥35 years old	180	23 (11.3)	114	23 (16.8)
Maternal psychiatric condition				
No	470	72 (13.3)	244	61 (20.0)
Yes	145	23 (13.7)	140	18 (11.4)
Maternal smoking during pregnancy				
No	582	85 (12.7)	339	69 (16.9)
Yes	33	10 (23.3)	45	10 (18.2)
Maternal asthma prior to child’s birth^d^				
No	431	66 (13.3)	271	53 (16.4)
Yes	142	26 (15.5)	98	22 (18.3)
Maternal allergy prior to child’s birth^e^				
No	313	59 (15.9)	212	45 (17.5)
Yes	262	31 (10.6)	157	31 (16.5)

^a^Percent exposed within POP controls

^b^Percent exposed within ASD cases

^c^Includes Asian, Hispanic (race not specified), multiracial, and all others

^d^45 POP and 19 ASD are missing information on maternal asthma prior to child’s birth

^e^45 POP and 18 ASD are missing information on maternal allergy prior to child’s birth

We assessed that 95 mothers (13.4 %) of POP controls, 79 (17.1 %) mothers of ASD cases, and 86 (12.4 %) mothers of DDs were exposed to any type of occupational asthmagen during the pregnancy period (Table 2, Supplemental Table 2). We did not see a significantly elevated odds of having a child with ASD in occupational asthmagen-exposed compared to non-exposed mothers (adjusted odds ratio (aOR): 1.39, 95 % Confidence Interval (CI) 0.96–2.02). The most common asthmagen exposure groups were latex antigens, highly reactive agents, and cleaning and disinfectant products. The direction of the associations by type of asthmagen was generally positive comparing ASD cases to POP controls, but in an inverse direction when comparing DDs to POP controls (Table 2, Supplemental Table 2). In sensitivity analyses where we classified mothers without pregnancy jobs as unexposed, the results were similar although the ORs for ASD were slightly attenuated (Supplemental Table 3). Among 177 ASD children without intellectual disability (ID), 26 (14.7 %) had a mother exposed to an occupational asthmagen during the pregnancy period compared to 53 of 279 ASD children with ID (19.0 %). Comparing asthmagen exposed to non-exposed mothers, the adjusted OR was 1.29 (95 % CI 0.75–2.19) for ASD without intellectual disability and 1.45 (95 % CI 0.95–2.21) for ASD with intellectual disability.

**Table 2:** Crude and adjusted odds ratios and 95 % confidence intervals for ASD comparing maternal occupational asthmagen exposed to unexposed

Exposure	POP (N = 710)		ASD (N = 463)			
	N	%	N	%	cOR (95 % CI)	aOR^a^ (95 % CI)
*Any asthmagen*	95	13.4	79	17.1	1.33 (0.96–1.84)	1.39 (0.96–2.02)
Any HMW	69	9.7	58	12.5	1.33 (0.92–1.93)	1.38 (0.91–2.10)
Latex	63	8.9	47	10.2	1.16 (0.78–1.73)	1.17 (0.75–1.84)
Any LMW	45	6.3	37	8.0	1.28 (0.82–2.02)	1.17 (0.69–1.98)
Reactive	34	4.8	29	6.3	1.33 (0.80–2.21)	1.32 (0.73–2.37)
Cleaning	20	2.8	18	3.9	1.40 (0.73–2.67)	0.87 (0.40–1.87)

*POP* population controls, *ASD* ASD cases, *cOR* crude odds ratio, *aOR* adjusted odds ratio, *HMW* high molecular weight, *LMW* low molecular weight

^a^Analyses adjusted for maternal race (white, black, Asian, Hispanic, multi-racial/other), maternal education (less than high school, high school, some college/trade, bachelor’s degree, advanced degree), current household income at time of questionnaire (<$30,000, $30,000–70,000, $70,000–110,000, $110,000+), maternal age at birth (continuous), parity (1, 2, 3 or greater), active smoking during pregnancy (yes, no), maternal psychiatric condition history (yes, no), and child’s sex

We saw no statistical evidence of heterogeneity of associations in models for which we calculated interactions. The point estimate for the association between occupational asthmagen exposure and ASD was higher among female children (aOR 2.00, 95 % CI 1.03–3.89, 64 cases) than among male children (aOR 1.20, 95 % CI 0.78–1.85), but based on a relatively small number of female children (*p* value for interaction = 0.2). The point estimate was higher among mothers with a history of allergy (aOR 1.90, 95 % CI 1.03–3.52) than among mothers without a history of allergy (aOR 1.09, 95 % CI 0.67–1.78) (*p* value for interaction = 0.2). When considering maternal history of asthma, the adjusted odds ratio was 1.54 (95 % CI 0.76–3.13) among mothers with a history compared to 1.28 (95 % CI 0.82–2.02) among mothers without a history of asthma (*p* value for interaction = 0.7).

## Discussion

In this multisite study with estimated maternal pregnancy occupational asthmagen exposure derived from detailed maternal job information, and a JEM designed specifically to assess occupational exposures to agents that trigger asthma, we did not observe an association between maternal occupational exposure to any occupational asthmagen and ASD. Though occupational asthmagen exposure differed by sociodemographic factors, we observed a result that was consistent with the null following adjustment for possible confounders. The direction of association was generally positive comparing ASD cases to POP controls and inverse comparing DDs to POP, but we do not have an explanation for these differences.

We hypothesized two possible mechanisms that could link maternal occupational asthmagen exposure to ASD in the children. First, maternal exposure to asthmagens may trigger a maternal immune response characterized by altered cytokine levels that may in turn affect neurodevelopment. Links between ASD and differences in cytokine levels in maternal serum during pregnancy (Goines et al. 2011; Jones et al. 2016) and amniotic fluid (Abdallah et al. 2013) have been reported. Animal studies also support the hypothesis that immune activity during pregnancy can influence brain development and behavior in the offspring (Meyer et al. 2009). Second, maternal occupational asthmagen exposure may trigger active asthma which might impact neurodevelopment through reducing oxygen availability to the fetus. One study found some suggestion of an association between hypoxia at birth and ASD among boys (Burstyn et al. 2011). A non-specific array of obstetric complications that are associated with fetal hypoxia have also been linked to ASD, leading Gardener et al. (2011) to hypothesize that fetal hypoxia may play a role in ASD. However, since we did not have measurements of maternal cytokine production or fetal oxygenation, our data did not permit explicit testing of these pathways from maternal exposure to asthmagen to ASD in offspring.

Two previous occupational studies examined certain categories of asthmagenic agents in relation to ASD. Windham et al. (2013) reported an association between maternal occupational exposure to disinfectants and increased risk of ASD, whereas McCanlies et al. (2012) did not find links between parental (maternal and paternal combined) occupational exposure to metals or disinfectants and risk of ASD. We did not find an association between maternal occupational exposure to cleaning/disinfecting products and ASD. Windham et al. (2013) obtained occupational information from birth certificates, whereas we based exposure assessment on occupational histories from detailed questionnaires.

Another important difference between our study and earlier studies is that we used an asthma-specific JEM while the two earlier studies used expert opinion to assess exposure. While both approaches have limitations, expert opinion is hampered by the degree of detail in the occupational histories and the familiarity of the experts with particular occupational settings (Teschke et al. 2002). We utilized a JEM that has been used to demonstrate associations between asthmagen exposures in the workplace and asthma (Kogevinas et al. 2007; Le Moual et al. 2004; Beach et al. 2012). Unfortunately, exposures assessed by a JEM could be misclassified for a variety of reasons, including the assumption that individuals with the same job codes have the same exposure, despite great variation in workplaces. The JEM also assumes that individuals are either exposed or unexposed to an agent, but in reality there is a gradation in the intensity of exposure. An advantage of this particular asthma JEM is that it includes built-in re-evaluation steps for certain ISCO codes to improve the exposure assessment. However, even with this advantage, the asthma JEM may have limited sensitivity (Liu et al. 2009; Beach et al. 2012).

Asthma is highly genetic in nature with some individuals showing allergic responses to agents that do not induce responses in other individuals (London and Romieu 2009). Given differential susceptibility to asthmagenic agents, we examined whether the association between asthmagens and ASD differed for those with and without a history of maternal asthma or allergy. While our results are consistent with no overall association between asthmagen exposures and ASD, the positive direction of the OR was largely driven by mothers with a history of maternal allergy prior to the child’s delivery. This could suggest a link between asthmagen exposure and ASD among allergic women, but it could also be a chance finding due to the small sample size, especially since we do not see any suggestion of the asthmagen effect being stronger in asthmatic compared to non-asthmatic mothers. Additionally, our maternal asthma and allergy variables represent a history of asthma or allergy and not a specific indication that these conditions were active during pregnancy or that asthma or allergic conditions preceded occupational asthmagen exposure. Without consideration of genetic background, it may be challenging to detect an association between maternal asthmagen exposure and ASD, if there is indeed heterogeneity in the association that is dependent on genetic vulnerability to asthmagens.

If maternal allergy or asthma is associated with ASD and women with asthma or allergy avoid jobs with asthmagen exposure, then we may underestimate the occupational asthmagen to ASD association, part of a phenomenon known in the literature as the healthy worker effect (Pearce et al. 2007). However, we do not have enough information from the data to assess if healthy worker effect is truly influencing study findings. We see some indication of reduced likelihood of maternal occupational asthmagen exposure in women with a history of maternal allergy, but not asthma, prior to the child’s birth, but it is not clear if this is an artifact of small numbers, confounding, or suggests that some elements of the healthy worker effect could be at play. We also found that occupational asthmagen exposure was associated with lower socioeconomic status. If women with lower socioeconomic standing need to work in certain positions regardless of health conditions, then healthy worker effect may not have a strong impact on our results.

We also examined whether or not the association between asthmagen exposure and ASD differed by child’s sex because of the higher prevalence of ASD among boys (Fombonne 2003) and suggestion of greater hypoxia effects on ASD in boys (Burstyn et al. 2011). There is also a body of literature suggesting differential susceptibility to environmental toxicants in male versus female fetuses (DiPietro and Voegtline 2015). Though the point estimate of the adjusted odds ratio for ASD was stronger for female children compared to male children, we do not see statistical evidence of heterogeneity by sex. This lack of significant interaction may represent lower power due to a relatively small group of female cases that could be addressed in future research.

Limitations of our study include that both occupational histories and health conditions were based on self-report, which may be subject to recall bias because women with a child with an ASD or a developmental disorder may recall health conditions or describe occupational tasks differently than mothers of typically developing children. We estimated occupational asthmagen exposure based on a JEM since we did not have any direct measurements of exposure. We also recognize that fathers could bring home asthmagen exposures from the paternal workplace and expose other family members (Krakowiak et al. 1999; Krop et al. 2007; Tagiyeva et al. 2012) and that there are non-occupational sources of asthmagen exposure, but we did not account for these additional possible exposure sources in our analyses. However, we suspect that direct maternal exposures would be more intense and sustained than indirect paternal exposures that might be brought home. We do not hypothesize any direct biological link between paternal asthmagen exposures and ASD that is not mediated through the mother, so we suspect that associations would be weaker for paternal compared to maternal exposures. We also did not account for differences in timing of exposure during pregnancy and for potential differences in use of personal protective equipment. Selection bias into the SEED study is also a concern because maternal age, race-ethnicity, and education differ in the POP control group compared to the source population (DiGuiseppi et al. 2016). Unfortunately, we were unable to assess for the possibility of selection bias in our analysis because we did not have information on the occupational asthmagen exposure distribution in the source population. However we did control for these variables in adjusted analyses.

A major strength of our study over many others examining environmental and occupational exposures in ASD is the extensive phenotyping carried out in SEED. Experienced clinicians used the ADOS and ADI-R, current gold standard diagnostic instruments in ASD research, in a standardized and reliable method across sites to establish ASD classification. Research relying on less detailed phenotyping may have outcome misclassification bias. Additional strengths included detailed occupational history information, standardized application of an asthmagen specific JEM, the ability to consider a wide-array of potential confounders in the analysis, and large sample size compared to previous studies assessing occupational exposures in relation to ASD.

In conclusion, despite investigation in a large, multisite study with extensive ASD phenotyping and asthma-specific occupational coding, we did not find evidence for a measurable association between maternal occupational exposure to asthmagens during pregnancy and ASD in the children. Our focus on these occupational triggers does not rule out involvement of other maternal immune system triggers in relation to ASD.

## Electronic Supplementary Material

Below is the link to the electronic supplementary material.

Supplementary material 1 (PDF 91 kb)
