# Supplementary material for: Maternal Exposure to Occupational Asthmagens During Pregnancy and Autism Spectrum Disorder in the Study to Explore Early Development
Source: J Autism Dev Disord. 2016 Aug 10;46(11):3458–68. doi: 10.1007/s10803-016-2882-6 (PMC5073112; doi:10.1007/s10803-016-2882-6)
Supplement: Supplementary file 1 — Supplementary material 1 (PDF 91 kb) [file 10803_2016_2882_MOESM1_ESM.pdf]

Supplemental Table 1. Maternal occupational asthmagen exposure by characteristics among children with non-ASD developmental delays or disorders (DDs).

Characteristics	DD, N (% exposed)	
	Unexposed (N=607)	Exposed (N=86)
Child's Sex		
Female	211	23 (9.8) [a]
Male	396	63 (13.7)
Parity		
1	283	38 (11.8)
2	212	27 (11.3)
3 or greater	112	21 (15.8)
Maternal Race		
White	423	52 (10.9)
Black	103	20 (16.3)
Other [b]	81	14 (14.7)
Maternal education		
High school or less	71	9 (11.3)
Some college/ trade	152	45 (22.8)
Bachelor's degree	206	21 (9.3)
Advanced degree	178	11 (5.8)
Current Household Income		
<$30,000	109	21 (16.2)
$30,000 – 70,000	174	27 (13.4)
$70,000 – 110,000	160	22 (12.1)
≥$110,000	164	16 (8.9)
Maternal Age at Birth		
<25 years old	75	12 (13.8)
25-29 years old	128	30 (19.0)
30-34 years old	215	28 (11.5)
≥35 years old	189	16 (7.8)
Maternal Psychiatric Condition		
No	416	61 (12.8)
Yes	191	25 (11.6)
Maternal Smoking During Pregnancy		
No	549	75 (12.0)
Yes	58	11 (15.9)
Maternal Asthma Prior to Child's Birth[c]		
No	407	60 (12.8)
Yes	152	19 (11.1)
Maternal Allergy Prior to Child's Birth[d]		
No	325	50 (13.3)
Yes	233	31 (11.7)

[a] Percent exposed within DD controls
[b] Includes Asian, Hispanic (race not specified), multiracial, and all others
[c] 55 DD are missing information on maternal asthma prior to child's birth
[d] 54 DD are missing information on maternal allergy prior to child's birth

Supplemental Table 2: Crude and adjusted odds ratios and 95% confidence intervals for non-ASD developmental delays or disorders (DD) comparing maternal occupational asthmagen exposure to unexposed.

	POP (n=710)		DD (n=693)			
Exposure	N	%	N	%	cOR (95% CI)	aOR[a] (95% CI)
Any Asthmagen	95	13.4	86	12.4	0.92 (0.67 - 1.25)	0.85 (0.61 - 1.18)
Any HMW[b]	69	9.7	63	9.1	0.93 (0.65 - 1.33)	0.85 (0.59 - 1.24)
Latex	63	8.9	51	7.4	0.82 (0.56 - 1.20)	0.79 (0.53 - 1.18)
Any LMW[c]	45	6.3	38	5.5	0.86 (0.55 - 1.34)	0.69 (0.43 - 1.12)
Reactive	34	4.8	29	4.2	0.87 (0.52 - 1.44)	0.76 (0.45 - 1.29)
Cleaning	20	2.8	22	3.2	1.13 (0.61 - 2.09)	0.71 (0.37 - 1.38)

POP = population controls, cOR = crude odds ratio, aOR = adjusted odds ratio

[a] Analyses adjusted for maternal race, maternal education, current household income at time of questionnaire, maternal age at birth, parity, active smoking during pregnancy, maternal psychiatric condition history, and child's sex.

[b] HMW = high molecular weight

[c] LMW = low molecular weight

Supplemental Table 3: Crude and adjusted odds ratios and 95% confidence intervals for ASD and DD comparing maternal occupational asthmagen exposed to unexposed where mothers without a pregnancy job are classified as unexposed.[a]

	ASD vs POP		DD vs POP	
Exposure	cOR (95% CI)	aOR[b] (95% CI)	cOR (95% CI)	aOR[b] (95% CI)
Any Asthmagen	1.21 (0.88 - 1.66)	1.21 (0.85 - 1.73)	0.84 (0.62 - 1.14)	0.81 (0.59 - 1.12)
Any HMW[c]	1.22 (0.84 - 1.75)	1.25 (0.83 - 1.86)	0.85 (0.60 - 1.21)	0.85 (0.59 - 1.23)
Latex	1.07 (0.72 - 1.58)	1.08 (0.70 - 1.67)	0.75 (0.51 - 1.10)	0.78 (0.53 - 1.16)
Any LMW[d]	1.18 (0.76 - 1.85)	1.01 (0.61 - 1.67)	0.79 (0.51 - 1.23)	0.64 (0.40 - 1.02)
Reactive	1.22 (0.74 - 2.03)	1.14 (0.65 - 2.01)	0.80 (0.48 - 1.32)	0.73 (0.43 - 1.23)
Cleaning	1.29 (0.68 - 2.46)	0.79 (0.38 - 1.64)	1.04 (0.56 - 1.92)	0.61 (0.32 - 1.17)

ASD = ASD cases, DD= non-ASD developmental delay or disorder, POP = population controls, cOR = crude odds ratio, aOR = adjusted odds ratio

[a] Jobs for which we were unsure of overlap with pregnancy were excluded from theses analyses. Analyses include 647 ASD, 976 DD, and 921 POP.

[b] Analyses adjusted for maternal race, maternal education, current household income at time of questionnaire, maternal age at birth, parity, active smoking during pregnancy, maternal psychiatric condition history, and child's sex.

[c] HMW = high molecular weight

[d] LMW = low molecular weight
